# Ectopic Premolar Tooth in the Sigmoid Notch

**DOI:** 10.1155/2016/6426523

**Published:** 2016-07-28

**Authors:** K. Törenek, H. M. Akgül, I. S. Bayrakdar

**Affiliations:** ^1^Department of Dentomaxillofacial Radiology, Faculty of Dentistry, Ataturk University, 25040 Erzurum, Turkey; ^2^Department of Dentomaxillofacial Radiology, Faculty of Dentistry, Osmangazi University, Eskişehir, Turkey

## Abstract

Impaction of a mandibular premolar is relatively uncommon. Ectopic placement is more unusual and there has been no discussion in the literature of an ectopic mandibular premolar in the coronoid process. In this case report, we present an impacted ectopic mandibular permanent premolar in the sigmoid notch (incisura mandibulae) region. Etiology of the tooth and treatment options are discussed and illustrated by Cone Beam Computed Tomography (CBCT) images.

## 1. Introduction

Ectopic eruption is an anomalous situation in which the tooth does not trace its usual route [1]. Ectopic teeth have been occasionally discovered in unusual orientations or at a distance from their normal anatomic position, for example, in the orbit, maxillary antrum, nasal cavity, nasal septum, mandibular condyle, coronoid process, palate, and chin [2, 3]. Most mandibular molars, especially third molars, which are impacted far away from their original sites have been affected by a cyst or tumor. Only a few cases have been reported without associated cystic lesion [4].

In the present study, we report a case of an ectopic mandibular premolar located in the sigmoid notch with Cone Beam Computed Tomography (CBCT) that seems to have been displaced by neither cyst nor tumor.

## 2. Case Report

**Figure 1:**
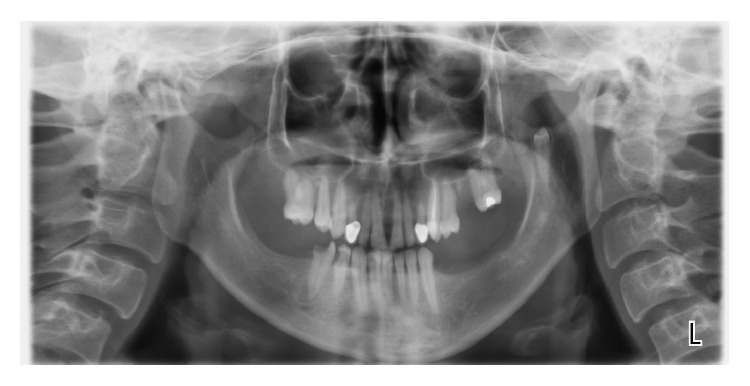
Panoramic radiograph showing an ectopic tooth located in the left mandibular ramus.

A 32-year-old female patient visited the Department of Dentomaxillofacial Radiology, complaining of pain in the right mandibular second premolar tooth. After intraoral examination, panoramic radiography showed an ectopic premolar located in the left mandibular ramus (Figure 1).

**Figure 2:**
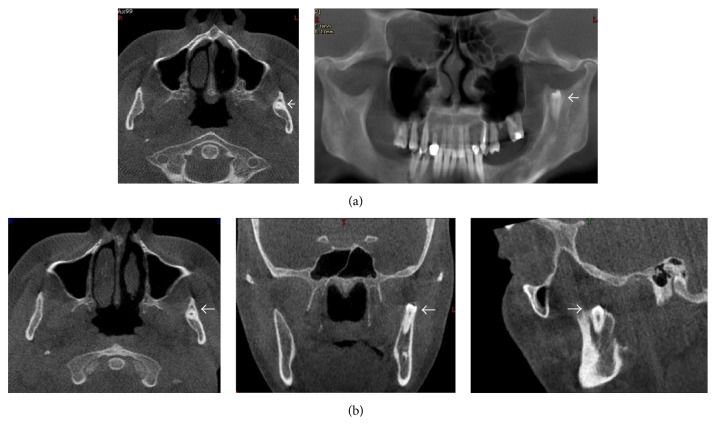
(a, b) CBCT scans show that the premolar is located in the sigmoid notch. (a) Panoramic radiography reconstructed by CBCT and (b) MPR-CBCT: (I) axial slice, (II) coronal slice, and (III) sagittal slice.

To identify the exact location of the tooth, Cone Beam Computed Tomography (CBCT) was taken, showing the premolar in the mandibular sigmoid notch in a vertical position (Figures 2(a) and 2(b)).

Surgical treatment was not seen as necessary for this patient because there were no symptoms related to the ectopic tooth. The patient was informed of the ectopic tooth and directed to clinical endodontics.

## 3. Discussion

Odontogenesis results from a complex multistep interaction between the oral epithelium and the underlying mesenchymal tissue. During development, abnormal tissue interactions may result in ectopic tooth development and eruption [5, 6]. The true incidence and etiology of ectopic eruption are still unknown, and researchers have suggested many theories, including trauma, infection, pathologic conditions, crowding, and developmental anomalies such as displacement of tooth buds. The displacement of tooth buds by the expansion of progressive cystic lesions, such as dentigerous cysts, may result in the dislocation of the tooth [7]. In the present case, displacement of the mandibular premolar was idiopathic.

Ectopic teeth are generally found in the jawbones or areas other than the alveolar arch, such as the nose, maxillary sinus, mandibular condyle, and coronoid process [8–10]. Symptoms, such as pain, generally occur in the mandibular coronoid and condylar regions. Asymptomatic cases are often discovered in routine clinical or radiographic examinations, as in the present case study.

Most ectopic teeth are third mandibular molars. Incisors and premolar teeth are present in the fewest ectopia cases. A review of the literature between 1980 and 2010 found 30 cases of ectopic teeth in maxillary sinuses, including 18 molars, 5 canine teeth, 3 supernumerary teeth, 1 odontoma, 1 tooth-like structure, and 1 premolar tooth [11]. In another review of the literature between 1965 and 2013, 25 cases of mandibular molars were reported, including 9 in the condylar regions, 8 in the subcondylar and ramus regions, 4 in the coronoid process, and 4 in the sigmoid notch [7]. A research of English-language studies showed 10 cases of ectopic mandibular second premolars, like this case report, including the condylar or subcondylar region from 1968 to 2010 [12].

In asymptomatic cases, ectopic teeth are not urgent; annual follow-up visits including panoramic radiography to display the anomaly are appropriate. In the symptomatic cases, the previous discussed studies, extraction of the ectopic tooth with the aid of endoscopy which represents an alternative to more demolishing traditional surgery allows a reduced postoperative morbidity and a shorter hospitalization period. Additionally, this technique offers to surgeons some advantages such as good illumination, clear visualization, and certain dissection [13–15]. Some surgeons have turned to endoscopic intraoral approaches; however, the routine use of endoscopy is expensive [7].

In this case report, an impacted ectopic premolar tooth was presented with conventional and CBCT images. Ectopic teeth have been observed on panoramic radiography generally, and there have been only a few reports of the use of CBCT. In summary, occurrence of an ectopic tooth in the sigmoid notch is rare and has an unclear etiology. There may be patients with an ectopic tooth without clinical symptoms, who do not know that they have a dislocated tooth. In these cases, the panoramic radiographs taken for different purposes are suggested for the early diagnosis of ectopic teeth.
